# Women’s experiences of life and healthcare after levator ani avulsion: a qualitative interview study

**DOI:** 10.1186/s12905-025-03892-z

**Published:** 2025-07-07

**Authors:** Karin Estendahl, Louise Danielsson

**Affiliations:** 1 Institute of Neuroscience and Physiology, Department of Health and Rehabilitation, Sahlgrenska Academy, University of Gothenburg, Gothenburg, Sweden; 2 SV Hospital Group Rehabilitation Centre, Alingsås Hospital, Alingsås, Sweden

**Keywords:** Levator Ani muscle avulsion, Birth trauma, Pelvic floor injury, Women’s health, Qualitative research

## Abstract

**Background:**

Levator ani muscle (LAM) avulsion affects approximately 10–20% of women following vaginal delivery and results in permanent damage to the pelvic floor. This condition is a significant risk factor for pelvic floor dysfunction, including pelvic organ prolapse (POP). Despite its high prevalence, we lack knowledge and clinical guidelines regarding the prevention, diagnosis, management, and treatment of LAM. Women’s views of living with this injury need to be acknowledged and explored.

**Aim:**

To explore women’s experiences of living with a LAM avulsion and their experiences of associated contact with healthcare.

**Methods:**

A qualitative interview study in which the data were analysed using qualitative content analysis according to Graneheim and Lundman. Fifteen women aged 33–55 years with diagnosed LAM avulsion participated. Videoconference interviews were conducted using a semi-structured interview guide with open-ended questions.

**Results:**

Three themes emerged: “Struggling to navigate healthcare”, “Restricting everyday life”, and “Seeing the future as uncertain, yet hopeful”. These themes highlight women’s frustration and challenges in navigating the healthcare system, their perception of a limited everyday life that must be adapted to what the body can handle, uncertainty about the future, and hope of good health and better care.

**Conclusions:**

LAM avulsion can substantially impact women’s quality of life. It is a hidden birth injury that needs attention. The results indicate many areas for improvement in healthcare, such as the value of confirmatory diagnosis and care that addresses the woman’s overall situation.

**Supplementary Information:**

The online version contains supplementary material available at 10.1186/s12905-025-03892-z.

## Introduction

Levator ani muscle (LAM) avulsion, i.e., the detachment of one or both muscle attachments from the pubic bone [1, 2], affects 10–19% of first-time mothers [3–7]. Factors during childbirth that can increase the risk of LAM avulsion include a prolonged second stage of labour, the use of forceps or vacuum extraction, and obstetric anal sphincter injury (OASI) [8, 9]. We lack reliable methods for predicting who is at risk of injury and how LAM can be prevented [10]. LAM avulsion affects the pelvic floor’s lifting and supporting function, increases the hiatus, and doubles the risk of pelvic organ prolapse (POP) later in life [11, 12].

Evidence-based surgical techniques to reattach a LAM that has detached from its attachment to the pubic bone are lacking, although some attempts at reconstruction have been described [13]. However, other parts of the birth canal, such as the perineal muscle attachments, which may also be damaged in a LAM avulsion, can be surgically reconstructed [10]. This may help mitigate symptoms such as the sensation of a wide vagina [14, 15]. Overall, there is a significant need for research to develop surgical interventions and to investigate whether other treatments, such as physiotherapy and vaginal pessaries, are effective to improve general POP symptoms or the sensation of vaginal looseness [13].

Few qualitative studies explore the patient perspective. An Australian study found that women who had suffered a LAM avulsion experienced reduced quality of life due to psychological and physical issues [16]. Symptoms of post-traumatic stress disorder (PTSD) were common. Furthermore, the study reported that healthcare providers seemed unaware of the consequences of this obstetric pelvic floor injury. The women described feeling traumatized and abandoned because the problems related to childbirth were discussed neither before nor after delivery.

Given the impact LAM avulsion has on women’s quality of life and the lack of treatment options, it is important to explore and improve preventative measures for avoiding injury [9]. Risk factors can be classified as those related to the woman, the child, and the obstetric management of labour. In the case of LAM avulsion, the risk factors appear to be similar to those for obstetric anal sphincter injury (OASI), such as instrumental delivery, labour augmentation with oxytocin, a second stage of labour prolonged over 60 min, and large infants [17]. At the same time, increased maternal age and low maternal body mass index (BMI) can also increase the risk [9, 18]. Studies have shown that measuring birth canal size, foetal head circumference, and pelvic floor elasticity can identify women at risk of avulsion for whom caesarean section may be preferred [19, 20]. Protective factors include warm compresses, perineal massage [21], and the presence of two midwives during delivery [22]. Recent initiatives such as OASI preventative care bundles outline antenatal and intrapartum strategies [23].

While there is no standardized rehabilitation for LAM avulsion, physiotherapeutic treatment for pelvic floor dysfunction may include pelvic floor muscle training to optimize the function of intact musculature, tone-regulating interventions for painful and tense pelvic floor muscles, general physical exercise, and advice and support regarding, for example, physical activity, ergonomics, and sexual health [24]. Other interventions, such as information, mechanical supports (e.g., vaginal pessaries), and psychological support, may also be valuable [10].

LAM avulsion is a relatively new diagnosis and is not widely recognized within healthcare, which may negatively impact the information and interventions offered to the afflicted women. To date, qualitative studies highlighting women’s voices are rare, mainly focusing on describing symptoms. To our knowledge, no study has explored how women with LAM-related symptoms perceive and cope with everyday life, what living with LAM avulsion means to them, or how they perceive the role of healthcare. This study accordingly explores women’s experiences of living with, and seeking healthcare for, symptoms related to LAM avulsion.

## Methods

### Design

A qualitative study was conducted using inductive content analysis according to Graneheim and Lundman [25]. Qualitative content analysis allows for the analysis of both manifest and latent content. The manifest content refers to the obvious, surface-level meaning of the text and is expressed descriptively. The latent content, in contrast, concerns the underlying meaning of the text and is expressed interpretively [25].

### Participants and recruitment

This study was conducted in Sweden and included women with a diagnosed LAM avulsion (unilateral or bilateral) verified by 3D/4D ultrasound. Reasons for exclusion were having an OASI as the primary injury or being unable to conduct a conversational interview in Swedish. The participants were recruited through advertisements on the blog *Bakingbabies* (written by a physiotherapist) and via physiotherapists in a national network in the field of women’s health. Seventeen women expressed interest in participating in the study: 15 responded to the advertisement and two were recruited through the network. They received written information and were asked questions to confirm eligibility. Fifteen women fulfilled the inclusion criteria and were accepted to participate.

Fourteen participants were interviewed via videoconferencing using Zoom and one participant was interviewed by telephone. All interviews were conducted by KE between 30 January and 6 April 2023. The interviews were audio recorded and lasted 22–63 min, with a median duration of 47 min.

The interviews were semi-structured, guided by an interview protocol with open-ended questions. The interviews covered the following topics: the path to diagnosis and current health, symptoms, work/leisure, sexual health, and rehabilitation/recovery.

A pilot interview was conducted to ensure that the questions were aligned with the study’s purpose. No changes to the interview guide were deemed necessary, and since the pilot interview provided valuable data for analysis, it was included in the study. The interviews were transcribed verbatim by KE on an ongoing basis during the data collection period.

### Data analysis

The analysis was conducted using qualitative content analysis according to Graneheim and Lundman, with a focus on identifying differences and similarities in the data [25]. First, the interviews were read in their entirety several times to get a sense of the overall context. After this, meaning units were identified and condensed to capture the core of their meaning. Each condensed meaning unit was assigned a code. The codes were compared and grouped into categories; see Table 1 below for examples of the process.

**Table 1:** An example of the process of creating meaning units, codes, and categories

Meaning unit	Condensed meaning unit	Code	Category
” And I’ve been dismissed time and time again. But I’ve always felt that something is wrong, that something isn’t right… [Sighs]”	I’ve been dismissed, but I feel something’s wrong.	Not taken seriously	Varying attitudes among healthcare professionals
“So, I guess you get a confirmation that there is actually a reason why I feel and have felt the way I do, and that it’s not in my head, but it’s in my genitals, and it’s broken.”	Get confirmation that there is a reason for how I feel; it’s not in my head, but in my genitals, and it’s broken.	Confirmation	The path to a confirmatory diagnosis

The first author (KE), in collaboration with LD, performed the analysis. Five transcripts were read and coded independently by both authors, after which the coding was discussed in detail. KE then coded the remaining transcripts. The manifest content was expressed at a descriptive level in the form of categories [25, 26]. The latent content, which refers to the underlying message of the text, was expressed at an interpretive level in the form of themes.

The first author’s (KE) preconceptions are based on personal experience with LAM avulsion, as both a patient and a healthcare provider. For several years, KE has met and treated women with pelvic floor-related issues as a physiotherapist in primary care. Such a preunderstanding can be helpful in perceiving nuances in the participants’ narratives, but it also needs to be made explicit to account for personal values and preconceived notions [27]. LD has a clinical and research background in mental health physiotherapy and chronic pain rehabilitation, which provided a different interpretative perspective. Throughout the analytical process, KE and LD held joint discussions and reflections to enhance the validity of the emerging results.

## Results

The participants came from different parts of Sweden, and there was variation in how long the women had lived with their LAM avulsion. They were 33–55 years old, with a median age of 39 years. The time from injury to diagnosis ranged from a few months to 26 years, with a median time of six years. The women had either unilateral or bilateral LAM avulsion. During the interviews, two participants revealed that, in addition to their primary diagnosis of LAM avulsion, they also had an OASI. While the interviews focused on the women’s experiences related to their LAM avulsion, their narratives illustrated complex lived experiences, suggesting that other potentially coexisting pelvic floor or postpartum conditions may be intertwined in their overall experiences. The participants had one to four children, with a median of two children. Fourteen of the women had higher education. Background characteristics of the participants are shown in Table 2 below.

**Table 2:** Background characteristics of the participants

	Participants *n* = 15
Age, years	
30–40	8
40–50	6
> 50	1
**Time between injury and diagnosis**,** years**	
< 1	2
1–5	4
6–10	5
11–15	2
> 15	2
**Diagnosis**	
Unilateral avulsion	4
Bilateral avulsion	11
**Healthcare level where diagnosis was made**	
University hospital	13
Other hospital/clinic	2
**Number of children**	
One	5
Two	8
Three	1
Four	1
**Education level**	
High school	1
College/university	14

The analysis revealed three themes: *Struggling to navigate healthcare*, *Restricting everyday life*, and *Seeing the future as uncertain*,* yet hopeful.* These themes reflect the implicit, latent meanings of the women’s narratives: their frustration and struggle to receive help in the healthcare system; a diminished and restricted daily life that must be adapted to what the body can manage; and uncertainty about the future, with glimpses of hope of restored health and better care. Each theme contained four categories, as illustrated in Table 3 below.

**Table 3:** Themes and categories identified in the analysis

THEME	Struggling to navigate healthcare	Restricting everyday life	Seeing the future as uncertain, yet hopeful
**CATEGORIES**	The path to a confirmatory diagnosis	Physical and mental well-being	Health care needs to do better
	Lack of knowledge persists	Impact on work and family planning	Women should be made aware
	Varying attitudes among healthcare professionals	Altered self-image and sexuality	Multidisciplinary support requested
	The pros and cons of surgery	Adapted physical activity	Thoughts about the future

### THEME 1: struggling to navigate healthcare

This theme highlights the women’s experiences of the healthcare system, a journey characterized by lack of knowledge, different responses from healthcare providers, surgical interventions, and, eventually, a confirmatory diagnosis.

#### The path to a confirmatory diagnosis

For the women, the journey through healthcare began in the first year after childbirth, often starting with a vague sense that something was wrong. They experienced and sought care for symptoms such as urinary incontinence, difficulty passing stool, a sensation of a vaginal bulge, pain, discomfort while walking, and a sensation of heaviness. For some, the symptoms, such as difficulty holding urine or contracting the pelvic muscles, became more apparent after a few years. One woman sought care eleven years after the injury due to an incident that suddenly triggered symptoms.

The path to diagnosis was described by the women as long and uncertain. They had to take an active and driving role in the process and felt that they themselves needed to seek healthcare and navigate the process of obtaining a competent and accurate assessment. During the interviews, it was revealed that many women sought healthcare outside their home regions through self-referrals. One participant sought care in five European countries before moving to Sweden. The women also described being “bounced around” between different healthcare providers, with long waiting times ranging from months to years:

And by then, I’d had 283 healthcare contacts, that is, visits, just for my vagina. I’ve had numerous conversations, and it makes me sad when I realize how much time I’ve spent on this. Everything has revolved around this, every week. But that’s how it is– it’s my healthcare journey. (Interview no. 15)

I’ve sought care several times since I got injured. I had symptoms from day one, quite pronounced symptoms, but never got them confirmed, just a pat on the back. ‘Well, you need to do pelvic exercises, it’s important that you do pelvic exercises’ and yes… nothing more. (Interview no. 3)

The path to diagnosis was marked by experiences of not being acknowledged or taken seriously by healthcare providers. It was described as frustrating to always have to take the lead in the process of seeking help. Some participants shared that they “gave up” when their symptoms were not validated as having an underlying cause.

The journey to diagnosis involved emotions such as doubt, worry, hope, despair, guilt, shame, and loneliness. For some, not being believed resulted in anxiety and led them to question their own experiences, while simultaneously dealing with concerns about their physical symptoms. Participants also reported receiving conflicting messages because of differing assessments from various healthcare providers. One participant described feeling torn between hope and despair when one healthcare provider initially suggested a solution, only to, months later, hear that she had a LAM avulsion and that surgical correction was not an option. Participants had been advised by healthcare providers to perform pelvic floor exercises. They expressed feelings of guilt, either due to their inability to perform the exercises effectively or because the exercises did not alleviate their symptoms. Some described feeling isolated in their suffering, keeping their struggles to themselves out of shame:

I couldn’t have normal bowel movements, and I didn’t understand why, even though I pushed and pushed, but nothing would come out… I didn’t seek help for it… I Googled a lot… what’s wrong with me… is this how it’s supposed to be… ahh, it was really embarrassing… it wasn’t something you’d mention to anyone… except maybe my husband… but almost not even to him… at first… because it was so strange. (Interview no. 1)

I haven’t spoken openly about my incontinence, not even to my husband, because I’ve been so ashamed. (Interview no. 3)

The women in the study found it meaningful and helpful to receive a diagnosis of LAM avulsion, in order to understand their symptoms. It provided relief to have an explanation for the issues they had experienced for years. For some, the diagnosis confirmed what they had already suspected. One woman described how the diagnosis alleviated feelings of guilt about having caused the injury herself, as she finally received an explanation. The participants emphasized the importance of validation, describing it as liberating to be believed and to “be proven right”. At the same time, they also expressed feelings of grief, noting the emotional difficulty of receiving the diagnosis, particularly hearing about the prognosis:

But at the same time, knowing it’s a levator injury also means I know that no matter how much I do pelvic floor exercises, it won’t help. It’s not my fault that it turned out this way. That’s how I’ve felt before– that it was my fault for not doing enough pelvic exercises. (Interview no. 1)

Oh, oh, oh, so much, of course! To have someone validate what you’re feeling and confirm it. I mean, now we’re in a different place. Now there’s clarity that there’s no research showing you can reattach the levator.… So, psychologically, it’s huge! And physically, in the form of an operation [i.e., perineal reconstruction] to make life better. So, yes, it’s absolutely worth a great deal. (Interview no. 7)

It confirmed what I already suspected, but I still found it quite tough. And the prognosis isn’t exactly encouraging, so, yeah. I wouldn’t say it was the ‘final nail in the coffin’, but it wasn’t great. I didn’t think it was a relief or particularly positive. (Interview no. 11)

#### Lack of knowledge persists

While the women expressed an understanding that knowledge of levator injuries might have been limited in the past, they felt that awareness among healthcare providers remains generally low even today:

Most [within healthcare] have no idea what it means. They have to Google it. Incredibly few have any clue at all! (Interview no. 8)

But when I came to her [i.e., the midwife], she was the first to say, ‘There is something called a levator injury, it might be that you have one’. So, thankfully, I’ve happened to meet people who have some knowledge, but I feel that many haven’t. (Interview no. 10)

The participants had largely taken it upon themselves to acquire knowledge and understand their symptoms, often utilizing resources such as social media. For example, the blog *Bakingbabies* was mentioned as a valuable source of information:

I started reading more about birth injuries. I began to recognize myself in what she [i.e., the physiotherapist/blogger] wrote. (Interview no. 7)

For me, it was clear from the start that it was levator injuries. Not immediately, because I didn’t know about it at all back then, but once I read up on it, it became obvious to me that it was cystocele, rectocele, and levator injuries. (Interview no. 4)

The participants believed that their LAM avulsion or other muscular injuries that could be surgically repaired (e.g., a second-degree perineal tear) should have been detected earlier during postpartum checkups with midwives:

If midwives had known more four years ago, maybe I could have received help with an operation [i.e., perineal reconstruction] back then. (Interview no. 6)

Yes, and as I said, my midwife thought everything was fine. It absolutely wasn’t. It really wasn’t. Absolutely not fine. I mean, when I was examined, the doctor said, ‘I don’t even need to bring out the ultrasound– it’s obvious, it’s so clear’. And I mean, how many postpartum checkups with midwives are there? There are a lot. (Interview no. 11)

The interviews also highlighted disparities among different parts of the country in the healthcare professionals’ skills and expertise. In some cases, professionals in the participants’ home regions had “overlooked” their LAM avulsion or failed to identify grade 2 muscular tears that could have been surgically repaired. As a result, these women expressed a lack of trust in healthcare providers in their home regions. In their search for competent care, feelings of being ‘at the mercy of the healthcare system’ and in a subordinate position were emphasized in the narratives.

#### Varying attitudes among healthcare professionals

Interactions with healthcare providers who had the expertise to diagnose LAM avulsion were often described as professional and responsible. The same was true for providers who referred the participants to specialists capable of making the diagnosis. However, the women also told of encounters when they felt unheard, or the focus was on the appearance of their genitalia rather than their function. Some participants described experiencing dismissive or belittling attitudes:

She gave me coffee, she was empathetic and professional, and I appreciated that she was honest, even though it’s a difficult diagnosis. She told me what it was. The truth is the most important thing. (Interview no. 2)

And I had all the prejudices beforehand and, unfortunately, they were just confirmed in the form of comments like, ‘It’s completely normal to wet yourself if you’ve had a glass of wine, get a little tipsy, and then lose control’. So, that feeling. Very dismissive and no in-depth questions at all. (Interview no. 1)

#### The pros and cons of surgery

Throughout their healthcare journeys, the participants had experienced various surgical procedures such as prolapse surgery, perineal reconstruction, hysterectomy (i.e., removal of the uterus), and Tension-free Vaginal Tape (TVT), which is a surgery for stress urinary incontinence. Some women were waiting for a procedure or had recently undergone surgery. TVT surgeries were described as successful in reducing stress leakage, and perineal reconstructions (when performed correctly) alleviated symptoms. However, there were also experiences of the surgical intervention not helping or having to be repeated:

All the surgeries have left me scarred and all that, but having a complete perineum… just the feeling when I went to the bathroom, ‘Wow, the hole has shifted’ or the distance is different, and the feeling of having a grapefruit between my legs was gone. So that was really nice. (Interview no. 3)

It was a very extensive surgery [i.e., anterior plastic surgery and perineal reconstruction], so I had pain for about a year afterwards from it, but it didn’t help with the actual problem, except that… well, things don’t fall out quite as much now. They’ve closed it a bit. But prolapse rings, you know, still stick out. (Interview no. 4)

### THEME 2: restricting everyday life

This theme highlights the women’s experience of having to hold back and adapt their bodies and lives in various ways after the injury. Limitations in daily life and both physical and psychological stresses impact family life, work, self-image, and sexuality.

#### Physical and mental well-being

In the interviews, participants described the initial postpartum phase as particularly challenging. They reported feeling battered, broken, as if they had been run over, and physically uncomfortable when walking or moving. Recovery was described as prolonged, and many expressed a sense of limitation when comparing themselves with other new mothers. For some participants, reflecting on their experiences during this phase was emotionally challenging, and in a few cases, the narratives indicated the onset of postpartum depression:

I couldn’t go for walks with the stroller… [Note: crying and wiping away tears] it felt like everything was about to fall out, and I leaked urine, and I couldn’t walk without having to stop and pee. (Interview no. 3)

When I went for walks, I felt a bit unsteady, so to speak, in the pelvic floor. I had others in my mom’s group who were out jogging and running, and… at the same time, it was the first time I had given birth, so I thought it was normal. (Interview no. 13)

In some of the interviews, however, it emerged that the early postpartum period was not particularly difficult, and the participants described their recovery as positive, with early physical activity being possible.

The physical symptoms the women described were multifaceted and affected them to varying degrees. These bodily experiences were described both concretely and more vaguely. Symptoms associated with bulging vaginal walls and prolapse, such as bowel emptying issues, a sensation of heaviness or fullness, chafing, and urinary urgency, were described. Pain in the pelvic area, pelvis, and lower back was reported by nearly half of the participants, and some women felt that their bodily stability and balance were affected. Some described a constant sensation in the pelvic area, a mild discomfort, overstrained pelvic floor muscles, and occasional cramping. Others instead described a feeling of looseness, an open sensation in the pelvic area, or a sense of lack of support in the pelvis.

Participants also experienced bodily changes, such as the pelvic floor no longer responding to pelvic floor exercises as before, the inability to use tampons, difficulty bearing down during bowel movements, and changes in how the gluteal muscles were affected by exercise:

I have this feeling of looseness. And I can still feel fatigue in my back. Like when I stand in line or stand around socializing. (Interview no. 3)

I feel instability all the time. I feel like I have to, I don’t know, like squeeze my muscles or tense them in another way. (Interview no. 7)

The women expressed a sense of worry and uncertainty related to the injury. They described holding back or limiting themselves in physical activities for fear of worsening their symptoms. This had consequences in family life, such as feeling less spontaneous or playful with their children:

Running after my kids, I’m very scared to do that, like when they’re at the playground and I have to run after them… then I’m nervous that it will get worse. (Interview no. 1)

I don’t run anymore. I used to do that a lot. Partly because I’m afraid. No one has told me I can’t, but I’m just too scared. (Interview no. 11)

At the same time, some women said that they did not feel as limited as they had feared, or that despite certain symptoms, they did not allow themselves to be held back by fear or anxiety when it came to physical activities:

Six years later, I can’t say it’s really affected me all that much. I thought it would have had a bigger impact. But I don’t know what will happen as I get older– there’s also a risk there. (Interview no. 9)

Thoughts about the injury occupied much of their daily lives. The participants expressed concern about the future and both sorrow and frustration over the relatively significant impact the injury had on their daily routines. This was expressed in various ways, such as distancing themselves from activities such as walking with friends, or dedicating more time to work because previously enjoyable exercise could no longer be done.

Mental health issues were also evident in the interviews. Affected participants made connections to the injury, such as exhaustion from not receiving validation from healthcare providers when seeking help, PTSD from birth trauma, and depression combined with physical discomfort. Two participants even described feeling suicidal, although they had received help with these feelings:

I was already tired before, and then the mental strain of waiting so long and not knowing who to talk to, and then when I sought help, and they said it was normal… that’s when I crashed. After 2021, I burned out. I was put on sick leave for exhaustion syndrome. (Interview no. 1)

For me, it feels like I can’t experience joy at all since I got this. And running meant so much to me– it was my way of coping when I felt bad, and it would make me feel better– and even though I try other things, I just… (Interview no. 4).

#### Impact on work and family planning

The interviews revealed that LAM avulsion often had consequences for the women’s professional lives, including sick leave, transitions to part-time work, the need to change jobs, reassignment, or adaptation of their roles to enable continued employment. The participants described various modifications, such as working from home part- or full-time, alternating between standing and sitting positions, altering job responsibilities, and adjusting night shifts. Professional setbacks were also noted, particularly in cases in which physical limitations prevented women from performing tasks associated with their prior areas of specialization. Additionally, the need for frequent bathroom breaks disrupted work routines, leading to unwanted interruptions, increased planning demands, and heightened stress. However, some participants reported no restrictions or changes in their professional lives as a result of the injury:

I work in an office, which means sitting quite a bit, and I get cramps in my gluteal muscles… Right now, I’m only on 25% sick leave, so, but I mostly work from home. Before the surgery, I managed because we’re allowed to work from home quite a lot. We have this agreement, so I’ve managed my work because I have a non-physical job. But if I had worked as a nurse or something, I wouldn’t have been able to work. (Interview no. 14)

Some interviews also revealed that the LAM avulsion impacted family planning:

It’s sad. It is. It affects the idea of having children. This is my first child. I would have liked to have more, but I don’t know if that’s going to happen. (Interview no. 11)

Now, I become extremely sad [Note: starts crying], but I really don’t think there will be more children. Because of everything that’s happened. It has been tough, both mentally and physically. (Interview no. 14)

I have to learn to live with it. I’ve had to accept that I probably won’t have more children. Even though I hadn’t planned that before, I feel that this injury made the decision for me– if you know what I mean. And that can make me feel sad sometimes, that I had to go through this kind of injury just because I wanted to have children. (Interview no. 10)

#### Altered self-image and sexuality

The interviewees described an altered self-image related to the LAM avulsion. Expressions of feeling old, broken, handicapped, unfeminine, and less beautiful were used by participants when describing their bodies and genitals. Participants who had identified as physically strong, enduring, and athletic before the injury felt they had lost a significant part of their identity. For example, they could no longer pursue their athletic ambitions, lead fitness classes as before, or keep up physically with male colleagues. Feelings of inadequacy as mothers and doubts about their abilities were also expressed:

I miss it, letting myself go, pushing myself, like a bit of speed and that sort of thing. Now it’s very controlled. So, I would say the hardest part for me has been that a large part of my identity was tied to being physical. And I can’t do that anymore. (Interview no. 13)

I feel like I’m ninety years old. I’m sad. I’m tired of life. Because I have this desire to move a lot [Note: becomes sad, teary-eyed], to work, dance, run, stand on my legs. But my whole life, since I was twenty-three, twenty-four, I’ve lived a very sedentary lifestyle, and I’m tired of it. So my whole body feels old. (Interview no. 2)

Enormous! I’ve lost my entire identity. Uh… because my identity was just being well-trained and sporty, I used to do many sports. (Interview no. 4)

It affects the rest of my body. If I don’t feel beautiful down there, because, I mean, a levator injury also involves… how should I put it… a visible change in the appearance of the genitals. (Interview no. 7)

The changed self-image and the physical symptoms also influenced the participants’ sexuality, but to varying degrees. They described problems with intimacy due to pain or discomfort in the genitals, altered sensation, pelvic floor weakness, feelings of looseness, air entering the vagina, and disturbances from bowel movements. Some participants described an unchanged or even improved ability to orgasm, while others had a decreased ability. Not all participants had reflected on their ability to orgasm, or had ever experienced orgasm, either before or after the injury. Pain in the genital area led some women to avoid penetrative sex or to engage in little or no sexual activity within their relationship, less than they desired. Some reported reduced interest in sex, with their sexual desire being affected. There was a sense of vulnerability in feeling damaged, experiencing a sensation of a wide vagina, and feeling less attractive in the genital area, which contributed to a view of themselves as “wounded” sexual beings. This sexual issue also sometimes affected the relationship with their partner. However, many said that their partner was understanding and supportive, and that the barriers were instead within themselves:

I can have sex, and it feels good. So, the function is good. But there’s just a little vulnerability, feeling like you’re a bit damaged, a bit wide, you know. (Interview no. 13)

And for me, who’s often in pain, I almost never feel like it. I’m in pain as it is, and no, I can’t even think about it. We have a very poor sex life. It doesn’t have to be penetration, but just becoming aroused causes more pain. (Interview no. 15)

Well, it still feels better in some way, or not worse– I don’t know if it’s because I… feel more confident now in sexual situations– but at least there’s no negative change. (Interview no. 6)

I just can’t, I don’t know why, not the same… uh, type of orgasm, like. So yes, it does affect that. Uh, I don’t know, I refuse to give in to it, but somehow, it’s just not the same anymore. Especially the feeling of looseness is quite bothersome. You always feel it, and you know that in certain positions, air comes in… so it’s definitely worse. (Interview no. 10)

#### Adapted physical activity

The women described having extensively adapted their daily lives to accommodate their pelvic floor. When pain was a dominant symptom, they made large adjustments to compensate in daily life. These women learned to handle daily tasks (e.g., laundry, cleaning, cooking, and dishwashing) by breaking them down, simplifying them (e.g., using a robot vacuum, hanging laundry while sitting, getting help, and sitting on something soft), and adapting them (e.g., adjusting walking distances and varying positions: sitting, standing, walking, and lying down). They also scaled back on physical recreational activities to avoid strain on their pelvic floor:

I am always thinking about how I should do things now so that it will cause the least discomfort and [I’ll] feel less pressure, regardless of whether I’m making porridge, hanging laundry, or walking the kids to school. (Interview no. 12)

The participants had changed the way they exercised, and some were less physically active than they wanted to be. Activities that were avoided included running, dancing, heavy strength training, soccer, and various fitness classes (e.g., aerobics, cross-fit, and dance):

I don’t run, for example. So that’s a huge difference. I avoid certain types of exercise. I don’t dare to jump, or go to Friskis [i.e., a non-profit organization organizing exercise activities], or bounce around like that. So, it’s really annoying, of course. (Interview no. 11)

However, some women felt they could still train and engage in activities such as running, strength training, skiing, and climbing despite the LAM avulsion and were not afraid to try things out. They simply made minor adjustments, such as reducing the amount of jumping, squats, and lunges in the same session, or running depending on how the pelvic floor felt and where they were in their menstrual cycle:

I refuse to be too affected by it. But of course, you have to think a little about it. But I can still do everything I want. I can even jump and stuff like that, or I keep going until something happens, I think. (Interview no. 10)

The women who made more adjustments described how they changed their exercise routines because of the LAM avulsion, such as opting for home workouts instead of going to the gym, swimming, or doing gentler activities (e.g., core training and yoga). They expressed acceptance of these adaptations and were grateful for what worked. They also felt hopeful about being able to engage in other forms of exercise in the future by “building up the body” despite the LAM avulsion:

I’ve chosen not to focus on what I can’t do, but now I focus on what I can do instead. I don’t mourn what I can’t do because I’m so happy that I can walk, hike, do yoga, and today I can walk a five- or eight-kilometre loop around town without having to pee once, and that’s just fantastic. We go hiking in the mountains, and I get tired in my pelvic floor after a long day of hiking, but then I do a little yoga and relaxation, and it works. (Interview no. 3)

You just have to think like this. You have to try to think, okay, what types of physical activity can I do, and then I try to adapt to what I can do and be happy for that, you know. (Interview no. 8)

### THEME 3: seeing the future as uncertain, yet hopeful

This theme highlights the women’s message to the healthcare system regarding potential areas for improvement in preventing LAM avulsions, identifying such injuries, and the support they wish to receive from healthcare services. While uncertainty influenced their thoughts about the future, they were hopeful about improved well-being, increased societal awareness, and better healthcare.

#### Healthcare needs to do better

Several participants believed that their injuries could have been avoided if maternity care had been better. These women felt that their births had been difficult and exacerbated by factors such as external pressure, not being listened to, or not receiving needed support. Issues included being denied a spot in the delivery room at an early stage, not having the option of a water birth, not feeling comfortable with the midwife, failing to assess the baby’s large size, the midwife not using perineal protection, or the induction making the birth feel forced:

It was the worst day of my life… even though my daughter, whom I love so much, was born that day, it’s… it’s horrible to think about. It hurt so much, and I felt like I got no help there either… I gave birth in Italy, but even there they kept trying to push me, they wanted to give me antibiotics, I didn’t want to take them, and it felt like everyone was against me. Maybe that made me even more tense. (Interview no. 4)

The participants reflected on whether the injury could have been prevented by a caesarean section, considering the baby’s size and position, the woman’s risk factors (e.g., low body fat, narrow pelvis, and age), or other external factors such as induction, use of a vacuum extractor, birth position, and collaboration with the midwife:

I’m not the expert here. But then it happened, I wasn’t advised ‘you should have a caesarean’, it was more ‘let’s try and see what happens’. Afterwards, I understood that it probably shouldn’t have been my decision to make. I mean, five kilos is quite a large baby, and… well, it could have happened anyway, but there were many factors that could have led to significant injuries. (Interview no. 14)

I’m not the expert here. But then it happened, I wasn’t advised ‘you should have a caesarean’, it was more ‘let’s try and see what happens’. Afterwards, I understood that it probably shouldn’t have been my decision to make. I mean, five kilos is quite a large baby, and… well, it could have happened anyway, but there were many factors that could have led to significant injuries. (Interview no. 14)

On the other hand, some participants described normal births that they experienced as good and uncomplicated:

They did everything they should have. He was a big baby. I don’t know– I don’t think there’s anything they could have done differently. I think it was very well handled. I’m very satisfied with the birth. (Interview no. 10)

I hope that, in the future, there’s more knowledge so that more injuries can be prevented. (Interview no. 6)

As previously mentioned, participants felt there was a lack of knowledge of pelvic floor muscles among midwives, resulting in muscle damage that could have been repaired, but was not properly stitched after the birth. Similarly, they reported that their injuries had not been discovered during their follow-up checks (with a midwife around six weeks postpartum), which they interpreted as a lack of competence. The women expressed the need for more thorough and longer-term follow-ups. They also wished that 3D ultrasound examinations had been offered earlier when they were seeking help for their problems:

The issue of getting support from the healthcare system is important. Most women turn to some childbirth injury group on Facebook to get information. You just sit and Google everything yourself. You Google and Google and Google. (Interview no. 5)

So, I wish they wouldn’t just let a woman with a weak pelvic floor go without further care. I don’t think any woman should have surgery for prolapse until she’s had a 3D ultrasound. ‘How do the muscles look?’ And not just do the ultrasound, but also interpret it. Because many hospitals do 3D ultrasounds, but they can’t interpret them. And if they can’t interpret them, there’s no benefit. These women who don’t respond as expected shouldn’t be dismissed. That’s my main wish. (Interview no. 3)

The participants highlighted the need for more structured and continuous care. Healthcare should provide written material about levator injuries, which the woman could read once diagnosed. Women also requested clearer guidance on where to turn if they experience increasing or new symptoms and wished for a follow-up plan. They pointed out that waiting times of over a year for assessments or surgeries were unreasonable:

Yes, it’s all about the aftercare after the diagnosis, in a way. Maybe providing brochures so we can read about it. Those are the things that I think are important– don’t leave women feeling so alone in these things. (Interview no. 10)

At first, I just wanted someone to believe me. From the very beginning, I wanted them to say, ‘Now we’ll find out what this is. We can’t see anything with the naked eye, but we will find out’.… I wish they had suggested it themselves. And that it wouldn’t take a year. There should be more clinics that do 3D ultrasounds, so I wouldn’t have had to wait for a year. And I wish they had maybe referred me to a physiotherapist instead of leaving it up to me to find one. (Interview no. 1)

#### Women should be made aware

The interviews revealed that the participants were unaware that they could suffer LAM avulsion during childbirth. The women described it as a hidden problem, that there are muscle injuries that cannot be surgically reconstructed and that can affect daily function. The participants wanted to raise awareness of LAM avulsion and its consequences, among both women and healthcare professionals. They claimed that if the injuries were paid more attention, there would be a chance for better care and rehabilitation:

But that’s exactly it. I was struck by this, you know, [that] the levator injury first got a diagnostic code in the medical records in 2020. Eh… [laughs] and you can figure out for yourself that levator injuries– I mean women have given birth vaginally before 2020, you know. Eh, and just this knowledge gap, I think there are so many women who don’t even know they have it. That you have something called levator musculature in the pelvic floor, you know. And it’s not even talked about, it’s just the tearing scale when talking about vaginal births. It’s grade one to grade four, like that’s it. (Interview no. 14)

Many women want children, and many know that childbirth can cause damage. I knew that too, in a way. But I didn’t know that it could be like this, with muscles that cannot be sewn back together at all. You think that most muscles can be stitched, but these cannot be fixed, yet. (Interview no. 10)

I think it’s really good to raise awareness that you can actually be so injured that you won’t fully recover, and that it can limit [you] a lot. But also, that there’s so much that can be done about it, if you’re just believed and discovered. (Interview no. 15)

#### Multidisciplinary support requested

The participants highlighted the need for more multidisciplinary care than is currently offered. For example, the women suggested that supportive counselling should be offered with a psychologist or counsellor. Unresolved birth traumas were described, with some participants believing that counselling at the time of diagnosis would have been helpful, and others expressing an ongoing need for support in managing their situation. Support and advice about sexuality should also be developed, and some women expressed a desire to see a sexologist in the future. Some participants requested opportunities to share experiences with other women affected by LAM avulsion, to feel less alone and gain constructive tips on how to improve daily life. Various suggestions were made, such as offering group therapy in the healthcare system, providing webinars, podcasts, and Facebook groups, and forming patient associations:

But I have not been offered any form of counselling support. I actually find that quite remarkable. There are often counsellors associated with women’s clinics. It’s so… maybe it’s not for everyone, but I think there was so much grief work, in some way. And I think a lot of people do experience that, even though they might not think… I think it should be offered. (Interview no. 11)

Yes, but some form of support on how to handle it, the whole thing, psychologically. Perhaps be part of a group in which people have similar concerns, you know. Yes… counselling help and maybe physiotherapy help, and perhaps something team based, something that supports you a little, you know. (Interview no. 5)

But I really believe in getting help with others who have the same… so that you can share, know that I am not alone in this.… There is so much to work on around it, it isn’t just the physical part here. (Interview no. 12)

Some participants had experience of physiotherapy related to their injury. They emphasized that all healthcare providers should know about the consequences of birth-related injuries and ask about this when, for example, women sought treatment for back or pelvic pain. The participants saw a need for more physiotherapists who could perform internal pelvic floor muscle examinations, both to detect missed muscle injuries that can be stitched and to identify LAM avulsion.

The participants requested support from a physiotherapist and would have appreciated being referred to someone with specialized knowledge in this field. Seeking care outside their region, having digital consultations with physiotherapists who had more expertise on LAM avulsion, and participating in a digital pelvic pain school were options for some. They described how physiotherapy could increase their hope of a good life despite the injury. They wanted guidance on physical activity and exercise, but also recognized the need to generate more knowledge on feasible exercise options for individuals with LAM avulsion. They hoped that potential links among muscular problems, pain, and levator injuries would be more researched in the future.

Several women also had experience of commercially available online rehabilitation programmes that had been helpful. Some participants described difficulties in accessing help to try a vaginal pessary and identified opportunities for improvement in how different types of support (for both incontinence and POP) are designed and implemented within the healthcare system:

I believe it’s helpful to have more specialized physiotherapists. Many are very generally skilled in many things. But this is an area that doesn’t seem to be well understood. So more investment in women’s health and in physiotherapists who are also skilled in performing vaginal examinations. (Interview no. 15)

I think the programme [purchased online] has been very helpful, as you get to do things with your body. When you do things with your body, you always feel better, and then your mind feels better. And that’s what has helped me feel good. (Interview no. 12)

#### Thoughts about the future

The women had mixed feelings when thinking about the future. Some expressed concern about how their symptoms would develop as their bodies aged. There was uncertainty about treatment options in case the injury led to POP that required surgery. However, some women believed in and hoped for improved care in the future with increased knowledge of the diagnosis. Many also felt hopeful about the possibility of improvement through rehabilitation and surgery:

I don’t know how it will affect me in the future. I don’t fully know what affects me right now either. (Interview no. 9)

But I have this rehab programme that I’m going to do. And I think I’ll really try to build up, from scratch, all the muscles around it and try to find a way to exercise with the levator injury, so that I can also get physical exercise for my well-being. And to keep my body functioning. (Interview no. 13)

## Discussion

This study shows that LAM avulsion can have profound consequences on multiple levels for affected women, particularly regarding their quality of life. It also highlights that LAM avulsion remains an under-recognized issue and that there are many areas for improvement within healthcare.

The women in our study revealed significant gaps in the care chain as they described their long journey to receive a diagnosis. A lack of knowledge, across different levels of healthcare, of LAM avulsion and its consequences has previously been noted [13, 28].

The results confirm well-known physical symptoms associated with LAM avulsion [10, 12] and add detailed descriptions of how the women in our study managed these symptoms through adaptations, particularly regarding physical activity. The findings indicate that most participants adjusted and limited their physical activity. The women felt insecure about how to engage in physical activity, expressing concerns about pelvic floor symptoms and a fear that daily activities or exercise would contribute to POP or exacerbate other symptoms in the long term. A similar pattern has recently been described among younger women with symptomatic POP [29, 30]. A recent Australian study reported that one in three women identify pelvic floor issues as the main barrier to exercising [31]. This is concerning given the WHO recommendations on physical activity and exercise to promote health and prevent physical and mental illness [32]. This points to a need for healthcare professionals to inform, guide, and support women when it comes to post-injury exercise. Avulsion can indeed lead to an increased levator hiatus and a weakened pelvic floor, which may explain the elevated risk of POP [12]. However, studies have shown that LAM avulsion is not the sole factor contributing to an increased levator hiatus. Other contributing factors are believed to include connective tissue characteristics in the perineal membrane and perineal body, which remain less well-studied [28]. Not everyone with LAM avulsion develops POP, and research on POP and physical activity in general is still limited [33]. For instance, one study found no association between POP and physical activity/exercise in middle-aged women [34]. Extremely heavy training, such as that undertaken by military paratroopers, was shown in one study to increase POP risk [35]. Nevertheless, physical strain is not the primary risk factor for prolapse; known risk factors, in addition to LAM avulsion, include multiple pregnancies, vaginal deliveries, age, and high BMI [36].

Moreover, our results build knowledge of the psychological consequences that can arise. Skinner et al. [16] found that women’s mental health issues could be seen as a serious consequence of a healthcare system that failed to acknowledge or recognize women’s somatic complaints following a LAM avulsion. Psychological symptoms such as anxiety, depression, exhaustion, PTSD, and suicidal thoughts emerged in our results, in alignments with these findings. Similar findings were also highlighted in a study of younger women who experienced symptomatic POP after childbirth [30].

The category Thoughts of the future highlights that the women had doubts and fears about the future, which seemed uncertain to them. The concept of “illness uncertainty” has been defined in the literature as a cognitive stressor, a sense of loss of control, and a perceptual state of doubt or not knowing what changes to expect over time [37, 38]. It also includes unpredictability regarding healthcare’s inability to predict what the future holds for the individual [39]. The women in our study described several of the factors related to illness uncertainty: ambiguity about the illness condition, complexity of treatment and the healthcare system, lack of information about the diagnosis and severity of the illness, and unpredictability regarding disease progression and prognosis [40].

Skinner et al. [16] highlighted, in addition to lack of knowledge, stigma as a barrier to seeking help; accordingly, the women in her study did not want the interviews to be recorded. In our study, however, the women were open to talking about their problems and did not hesitate to be recorded for the sake of research. This suggests cultural differences, or that the stigma surrounding childbirth injuries is, in fact, changing. Possibly, recent attention in the Swedish media to neglected women’s health issues, as well as governmental initiatives to promote equitable health, may have impacted the participants’ attitudes towards letting their voices be heard [41]. Further steps to decrease stigma should be explored and encouraged.

An important finding of our study was how LAM avulsion can impact women’s sexual health. The participants’ experiences captured the complexity of sex, desire, and orgasm, which were valued differently by the women: while some described sexual problems that affected their quality of life and were reasons to seek help, others were neutral or positive about post-injury sexual relations. A previous study showed that sexual function can be affected by increased vaginal volume and decreased pelvic floor contraction strength during orgasm [42]. Other dimensions of sexual function, such as sensation, arousal, and ability to orgasm, appeared to be less affected. In a Swedish study, partners of women with symptomatic prolapse and LAM avulsion (eight out of 13) were interviewed, revealing that the woman’s injury negatively impacted intimacy and sexual relations [43]. Since few studies have yet investigated the long-term consequences for post-injury sexual health, this is an area for future research.

Another important finding of our study is that LAM can have consequences for family planning, a factor also highlighted in the previously mentioned study [43]. Healthcare professionals need to be made aware that one possible consequence of the injury and prior healthcare management is that couples may decide not to have the children they had hoped for. This emphasizes the importance of being receptive and responsive to the need for psychosocial support.

The results highlight the shortcomings of healthcare in identifying, diagnosing, and treating issues related to LAM avulsion. The women also provided suggestions for improvement. A step in the right direction, aimed at improving healthcare for women, is the national guidelines for pregnancy, childbirth, and postpartum care published by the Swedish National Board of Health and Welfare in December 2023 [44]. These guidelines aim for more equitable care, strengthened continuity in the care chain, and improvements in postpartum care. The healthcare regions are recommended to develop guidelines on how to refer the patient for treatment of various issues that may arise and persist after childbirth. Multidisciplinary assessment, treatment, and rehabilitation should be available. The guidelines include some recommendations aligned with the improvements the women in this study considered important. For example, it is recommended that women should be examined by two professionals (i.e., midwives or doctors) immediately after childbirth to identify any further obstetric perineal tears. Early follow-up of physical and mental health and additional assessments for tears should be offered when needed. However, it is important to note that while the women in our study wished for earlier ultrasound assessments, the efficacy of early 3D/4D ultrasound in identifying LAM avulsion is not supported by research [6, 45]. Diagnosis immediately post-birth or at six weeks is considered premature, as it may lead to false-positive findings [6]. A definitive diagnosis is generally not established until one year postpartum.

The women in our study highlighted physiotherapy as essential for managing this lifelong disability. Physiotherapists can assess muscular function, promote and adapt physical exercise, and provide advice and exercises to help women restore function in muscle groups that may affect pain and prolapse symptoms. Facilitating physical activity for women despite pelvic floor symptoms is important for preventing other health issues [32]. Currently, we lack evidence both on pelvic floor training as a treatment for prolapse symptoms when LAM avulsion is an underlying cause [46] and on whether pelvic floor training can reduce levator hiatus [47, 48]. Rehabilitation, ideally involving a multidisciplinary team, can help women expand their coping strategies and equip them with tools to improve their quality of life despite the injury.

### Methodological discussion

A strength of this study was the variation among participants in terms of age, duration of living with LAM avulsion, type of injury (i.e., uni- or bilateral), and the time between injury and diagnosis. There was also geographic diversity in where the participants sought care. The sampling strategy resulted in a broad range of experiences and perspectives, which enabled the gathering of rich and varied data, increasing credibility [25, 26]. However, while all the included women had experienced symptoms of and were diagnosed with LAM avulsion, it cannot be ruled out that other pelvic floor problems or the overall postpartum experience might have influenced their narratives. This can be regarded as a limitation from a methodological point of view, but such blending of experiences is likely also common in the clinical population.

It emerged during the interviews that two participants, in addition to their LAM avulsion, had sustained an OASI. Since their interviews provided valuable data that enriched the analysis, we decided to include them. This might be regarded as a limitation, but these narratives reflected two contrasting perspectives, highlighting the critical importance of the early diagnosis and treatment of reparable injuries to prevent unnecessary suffering.

The number of interviews in a qualitative study depends, among other factors, on the study’s aim and the quality and richness of the material [26, 49]. Malterud suggested using the concept of “information power” to evaluate whether the number of interviews provides sufficiently rich material for analysis [49]. We found nuanced experiences in the data and recurring narratives across the interviews, suggesting that the 15 interviews were sufficient for the purpose and scope of this study. We also observed that the patterns of meaning within the emerging categories and themes stabilized after the 11th and 12th interviews. While subsequent interviews provided further details and examples, these were still aligned with and supported the initial interpretations. This process suggests that thematic saturation had been achieved, meaning that additional data did not lead to the emergence of new themes [50].

To enhance trustworthiness, the authors independently reflected on the meaning of the data [25]. The interviewer had prior knowledge, having treated women with pelvic-floor–related issues for several years and having personally sought care for symptoms later identified as LAM avulsion. While this familiarity may have facilitated the interview process, it also posed a potential risk of bias during the analysis. The preconceptions were managed through critical reflection and discussions with the second author throughout the research process [27]. We outlined our preconceptions at the start of the study, to be aware of them and to discourage quick, taken-for-granted interpretations. During the analysis, we also deliberately sought statements that challenged the emerging interpretation. To highlight the link between the raw data and our interpretation, we have illustrated each category with several quotations.

To increase dependability, consistency of data collection was ensured by using the same interviewer and the same interview guide throughout the interviews [25]. An initial challenge was a slightly unstable Internet connection during the first interviews, leading to some questions being repeated and certain answers requiring clarification when the audio cut out. Subsequent interviews proceeded without technical issues. The videoconference format facilitated the inclusion of participants from across the country.

While the videoconference interviews might have complicated the creation of a relational, trustful atmosphere, the format has been suggested to make it easier for participants to discuss sensitive topics [51]. Additionally, compared with telephone interviews, the videoconference format enabled the reading of emotional cues through observing facial expressions and gestures.

A limitation of this study is that the findings are primarily transferable to women who have received a formal diagnosis of LAM avulsion. This means that the participants had experienced symptoms significant enough to cause them to seek medical care. Consequently, women with LAM avulsion who are asymptomatic or have not sought care were not represented, and their perspectives remain unknown. As a result, other aspects of living with LAM avulsion may have been overlooked.

Most participants were informed of the study through an advertisement on the *Bakingbabies* blog on social media. This platform is likely to primarily reach younger women with an academic background and those who actively seek information through such channels. No women over the age of 55 years volunteered to participate in the study, which may be seen as a limitation concerning the transferability to older women, suggesting a focus for future research.

## Conclusion

This study shows that LAM avulsion can substantially impact women’s quality of life. The participating women described limitations in their daily lives, as well as physical and psychological strain resulting from the injury. Their coping strategies varied, and feelings of anxiety, uncertainty, and frustration were common. The findings highlight the need for a confirmed diagnosis and emphasize the importance of healthcare taking a holistic approach, ensuring access to counselling support when necessary.

## Electronic supplementary material

Below is the link to the electronic supplementary material.

Supplementary Material 1

## Data Availability

The datasets generated during the current study are not publicly available due to the sensitive nature of the data and the potential to identify participants from the raw data. The data (in Swedish) may be available from the corresponding author upon reasonable request.

## Abbreviations

LAM: Levator ani muscle
POP: Pelvic organ prolapse
OASI: Obstetric anal sphincter injury
