# Supplementary material for: Women’s experiences of life and healthcare after levator ani avulsion: a qualitative interview study
Source: BMC Womens Health. 2025 Jul 7;25:336. doi: 10.1186/s12905-025-03892-z (PMC12232683; doi:10.1186/s12905-025-03892-z)
Supplement: Supplementary file 1 — Supplementary Material 1 [file 12905_2025_3892_MOESM1_ESM.docx]

**Interview Guide**

**Diagnosis and Current Situation**

- When, in your opinion, did you sustain your levator injury?
- When and how did you receive your diagnosis?
- What has it meant to you to receive a diagnosis?
- How would you describe the current state of your pelvic floor health?
- What does the levator ani injury mean to you?

**Symptoms**

- How do you notice that you have a levator injury?
- Do you experience discomfort in your pelvic floor? If so, in what way?
- How do you perceive your body overall?
- What does good pelvic floor health mean to you?

**Work/Leisure**

- How is everyday life for you?
- Do your symptoms affect your ability to work?
- How is it for you to engage in leisure activities that are important to you?
- Are you able to be as physically active as you would like? Any limitations?
- Is there any type of exercise/physical activity that works better or worse for you?

**Sexual Health**

- Does the levator injury affect your sexual health? If so, how?

**Rehabilitation/Recovery After the Levator Injury**

- How was the recovery after the delivery during which you sustained the levator injury? Did you receive any help from healthcare?
- If you sought but did not receive support, please describe what kind of help you would have wanted.
- Looking back on your delivery, do you have any thoughts on how the levator injury could have been prevented?
- Looking ahead, how do you view your future considering your levator injury?
